# Predictive value of high sensitivity C-reactive protein in three-vessel disease patients with and without type 2 diabetes

**DOI:** 10.1186/s12933-023-01830-7

**Published:** 2023-04-20

**Authors:** Lei Guo, Haichen Lv, Junjie Wang, Bo Zhang, Yifan Zhu, Xiaoyan Zhang, Hao Zhu, Xuchen Zhou, Yunlong Xia

**Affiliations:** 1 Department of Cardiology, the First Affiliated Hospital of Dalian Medical University, Dalian, People’s Republic of China; 2 Department of Cardiology, Shengjing Hospital of China Medical University, Shenyang, People’s Republic of China; 3 Department of Radiology, Fuyang Hospital of Anhui Medical University, Fuyang, People’s Republic of China

**Keywords:** High-sensitivity C-reactive protein, Three-vessel disease, Diabetes, Coronary artery disease, Outcomes

## Abstract

**Background:**

Diabetes mellitus (DM) and atherosclerosis are multifactorial conditions and share a common inflammatory basis. Three-vessel disease (TVD) represents a major challenge for coronary intervention. Nonetheless, the predictive value of high-sensitivity C-reactive protein (hs-CRP) for TVD patients with or without type 2 DM remains unknown. Herein, we aimed to ascertain the long-term predictive value of hs-CRP in TVD patients according to type 2 DM status from a large cohort.

**Methods:**

A total of 2734 TVD patients with (n = 1040, 38%) and without (n = 1694, 62%) type 2 diabetes were stratified based on the hs-CRP (< 2 mg/L vs. ≥ 2 mg/L). Three multivariable analysis models were performed to evaluate the effect of potential confounders on the relationship between hs-CRP level and clinical outcomes. The Concordance index, net reclassification improvement (NRI), and integrated discrimination improvement (IDI) were calculated to assess the added effect of hs-CRP and the baseline model with established risk factors on the discrimination of clinical outcomes. The primary endpoint was major adverse cardiac and cerebrovascular events (MACCE).

**Results:**

The median follow-up duration was 2.4 years. Multivariate Cox regression analyses showed that the incidence of MACCE (adjusted hazard ratio [HR] 1.17, 95% confidence interval [CI] 1.01–1.35, p = 0.031) and all-cause death (HR 1.82, 95% CI 1.07–3.11, p = 0.026) were significantly higher in the diabetic group compared to the non-diabetic group. In the diabetic group, the incidence of MACCE (adjusted HR 1.51, 95% CI 1.09–2.10, p = 0.013) was significantly higher in the high hs-CRP group than in the low hs-CRP group; no significant difference was found for all-cause death (HR 1.63; 95% CI 0.58–4.58, p = 0.349). In the non-diabetic group, the prevalence of MACCE (adjusted HR 0.93, 95% CI 0.71–1.22, p = 0.613) was comparable between the two groups. Finally, the NRI (0.2074, p = 0.001) and IDI (0.0086, p = 0.003) for MACCE were also significantly increased after hs-CRP was added to the baseline model in the diabetic group.

**Conclusions:**

Elevated hs-CRP is an independent prognostic factor for long-term outcomes of MACCE in TVD patients with type 2 diabetes but not in those without type 2 diabetes. Compared to traditional risk factors, hs-CRP improved the risk prediction of adverse cardiovascular events in TVD patients with type 2 diabetes.

**Supplementary Information:**

The online version contains supplementary material available at 10.1186/s12933-023-01830-7.

## Background

The latest epidemiological report predicts that the global diabetes mellitus (DM) prevalence will rise to 10.2% (578 million) by 2030 and 10.9% (700 million) by 2045 [1]. It has been established that patients with type 2 DM often experience a significantly greater atherosclerotic burden and higher risks of in-hospital and long-term adverse outcomes after revascularization than nondiabetic patients [2, 3].

Complex coronary arterial disease (CAD) is more common in patients with diabetes. Three-vessel disease (TVD) has been identified in up to 30% of all patients referred for diagnostic angiography, representing a major challenge for coronary intervention [4]. Additionally, the risk of death associated with TVD is almost two times higher compared to single vessel disease, and TVD is an independent prediction for poor clinical outcomes [5].

Indeed, type 2 DM is considered to be a state of low-grade inflammation [6–8]. Furthermore, current evidence suggests that chronic inflammation plays an important role in the pathogenesis of atherosclerotic CAD [9]. Type 2 DM and atherosclerosis are multifactorial conditions and share a common inflammatory basis. Furthermore, TVD patients exhibit a more prominent inflammatory state [5]. Recently, the presence of low-grade inflammation has been detected using high-sensitivity C-reactive protein (hs-CRP) [10]. A few studies have hitherto reported an association between elevated hs-CRP and cardiovascular events in patients with type 2 diabetes [7, 10, 11], however, the predictive value of hs-CRP for TVD patients with or without type 2 diabetes remains unknown. Therefore, in the present study, we sought to ascertain the long-term predictive value of hs-CRP in TVD patients according to type 2 DM status from a large cohort in a ‘real world’ setting.

## Methods

### Study population

This was a retrospective cohort study. Between January 2013 and December 2018, a total of 13,890 patients underwent coronary angiography at our institute. Patients are admitted to hospital for coronary angiography on the basis of the presence of symptoms, findings of ischemia by electrocardiogram, coronary computed tomography angiography, echocardiogram or other functional imaging tests. Patients with TVD were divided into diabetic group or non-diabetic group. Based on hs-CRP levels at admission, each study group was divided into two groups: Group 1, hs-CRP < 2 mg/L, and Group 2, hs-CRP ≥ 2 mg/L. A hs-CRP value ≥ 2 mg/L was considered a sign of inflammation, consistent with the current guideline [8]. Demographic data and procedural characteristics were collected from the institutional database. Follow-up data was obtained through the medical records, telephone contact interviews or outpatient visit. The study was approved by the local Institutional Review Board and informed consent was exempt by the committee.

### Inclusion and exclusion criteria

**Fig. 1:**
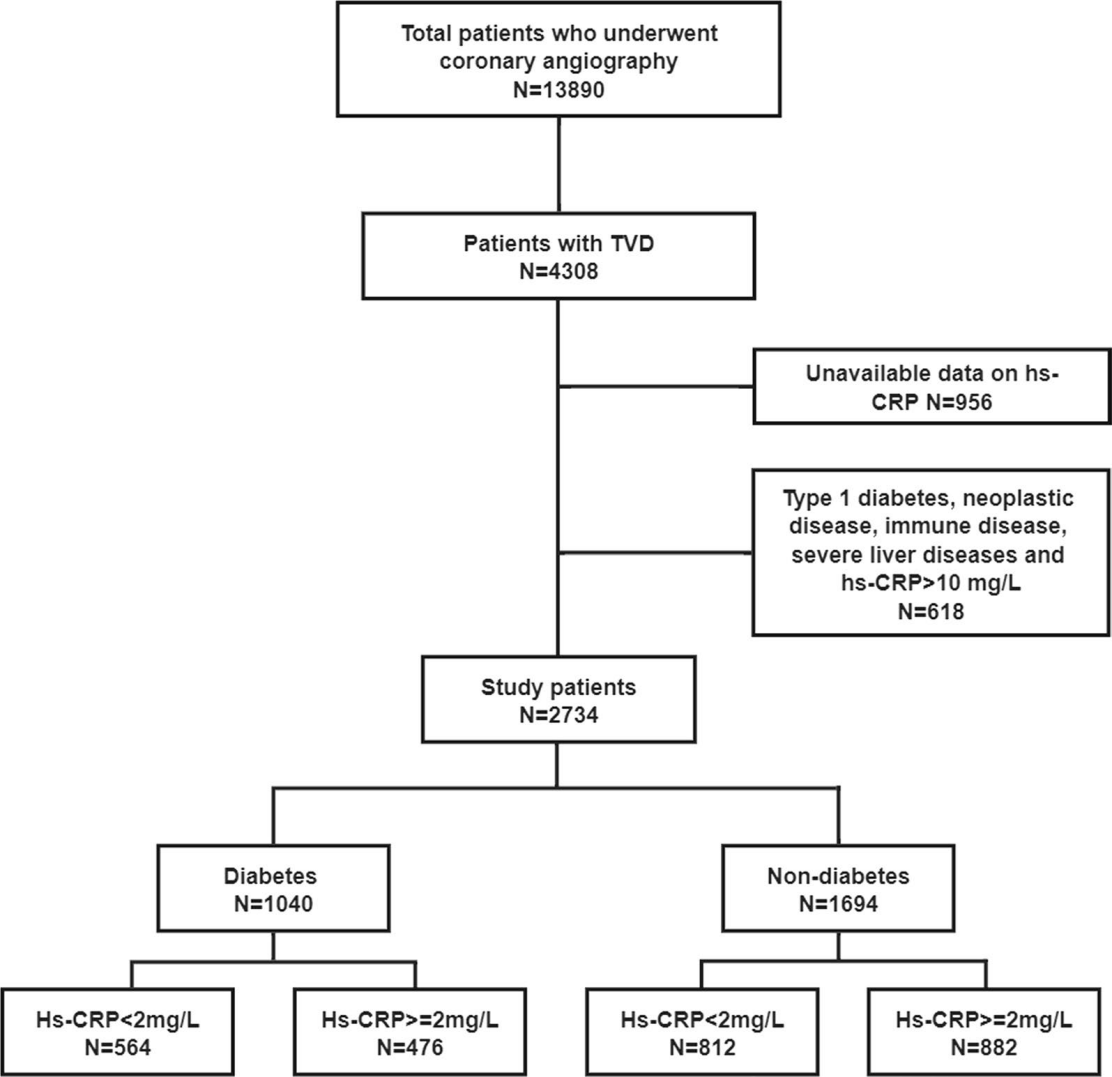
Study flow chart. *Hs-CRP* high-sensitivity C-reactive protein, *TVD* three-vessel disease

The inclusion criteria included patients ≥ 18 years of age and with TVD confirmed by coronary angiology. The exclusion criteria included out-patients, patients with type 1 diabetes, unavailable data on serum hs-CRP at baseline, neoplastic disease, immune disease, severe liver diseases, or inflammatory signs suggestive of active infection (hs-CRP > 10 mg/L). Figure 1 shows the study flow chart.

### Medical treatment and intervention procedures

Patients received medical therapy (MT) alone or revascularization according to current guidelines, interventional cardiologists’ judgment, and patient preference. Revascularization includes percutaneous coronary intervention (PCI) or coronary artery bypass graft surgery (CABG). Prior to PCI, all patients were pre-treated with loading doses of aspirin (300 mg) and clopidogrel (300–600 mg) unless they were previously medicated with these antiplatelet agents. PCI was performed in accordance with current guidelines and using conventional techniques. During the procedure, all patients were treated with heparin to maintain an activated clotting time of > 250 s. After the drug-eluting stents (DES) procedure, clopidogrel (75 mg/day) was prescribed for at least 12 months, and aspirin (100 mg/day) was continued indefinitely [12].

### Study definitions and endpoints

Serum hs-CRP levels were measured by the high-sensitivity nephelometric method (Dade Behring, Inc., Newark, DE, USA) at hospital admission. In this study, patients suspected of TVD underwent angiography to confirm stenosis ≥ 50% in three major coronary arteries (left anterior descending, left circumflex, and right coronary arteries), with or without involvement in the left main trunk (LMT).

Stable CAD was defined symptomatic patients with stable angina (chest pain on exertion for > 3 months) and asymptomatic patients with positive functional tests and obstructive coronary disease (defined as diameter stenosis > 50%). Acute coronary syndrome (ACS) includes unstable angina (UA), non-ST-elevated myocardial infarction (NSTEMI) and ST-elevated myocardial infarction (STEMI). NSTEMI and UA were defined by the presence of ST segment depression or T-wave abnormalities or ischemic symptoms with (NSTEMI) or without (UA) elevation of cardiac enzyme levels above the reference range. STEMI was defined by characteristic symptoms of myocardial ischemia in association with persistent electrocardiogram ST elevation and consequent release of biomarkers of myocardial necrosis [12]. Hypertension was defined as history of hypertension and the use of antihypertensive medication. Chronic kidney disease (CKD) was defined as an estimated glomerular filtration rate < 60 mL/min/1.73 m^2^. Severe liver disease was defined as transaminase levels ≥ 3 times the upper limit of normal. We defined diabetes mellitus as either hemoglobin A1c ≥ 6.5%, medication with oral hypoglycemic drugs, or insulin injections [13].

The primary endpoint of interest was the composite of major adverse cardiac and cerebrovascular events (MACCE), defined as all-cause death, repeat revascularization, myocardial infarction (MI), stroke, and readmission for angina pectoris or heart failure. The secondary endpoint was all-cause death. All-cause mortality was defined as death for any cause. Repeat revascularization was defined as a repeat PCI or bypass surgery. “MI” was defined as an increase in the concentration of creatine kinase-MB fraction or troponin-T/troponin-I greater than the upper limit of normal with concomitant ischemic symptoms or electrocardiographic findings indicative of ischemia. Readmission for angina pectoris or heart failure was defined as re-hospitalized in our hospital or other hospitals due to angina pectoris, acute heart failure or acute exacerbation of chronic heart failure [14].

### Statistical analysis

**Fig. 2:**
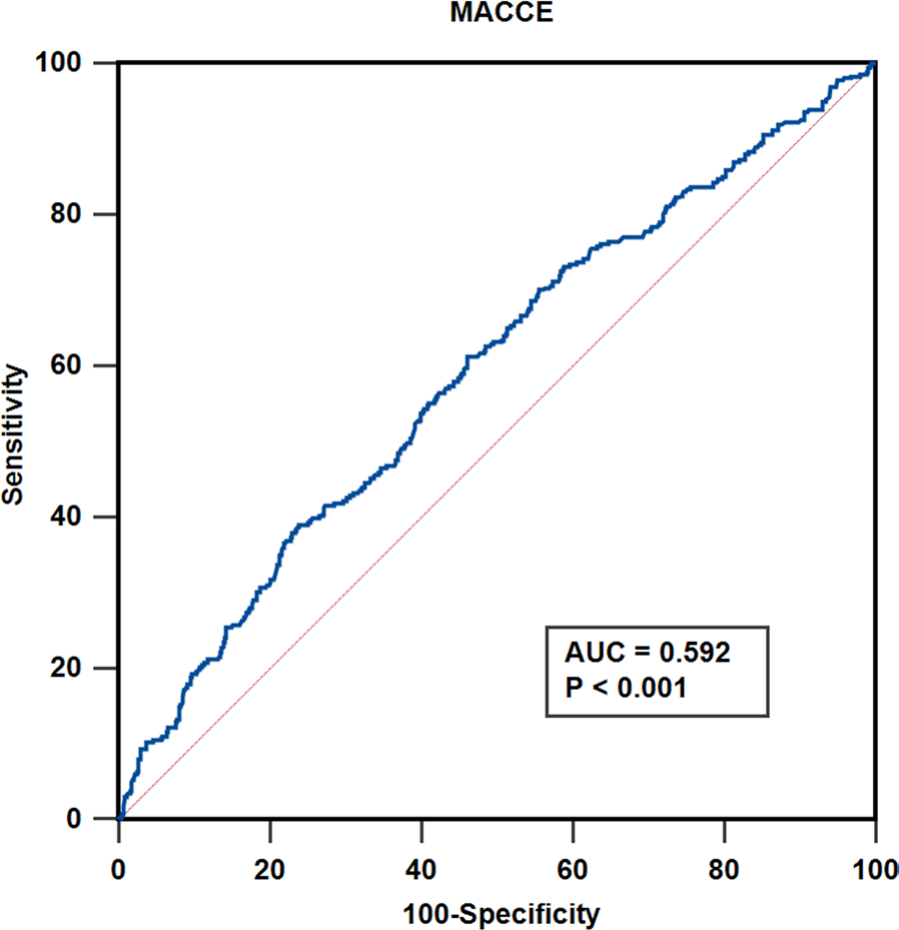
A ROC curve analysis to evaluate the predictive value of hs-CRP for MACCE. *AUC* area under the curve, *Hs-CRP* high-sensitivity C-reactive protein, *MACCE* major adverse cardiac and cerebrovascular events, *ROC* receiver operating curve

Categorical variables were compared with the chi-squared test and were expressed as percentages. Continuous variables were expressed as mean ± standard deviation or median (interquartile range), and were compared using the Student’s t test or Mann–Whitney U test. Event-free survival was estimated using Kaplan-Meier survival curves, and compared with the log-rank test. Three multivariable Cox regression analysis models were performed to assess the effect of potential confounders on the relationship between hs-CRP level and clinical outcomes. Independent baseline variables with a P < 0.1 in the univariate analysis and the relevant clinical implications were included in the multivariate models. Model 1 was adjusted for age and sex; Model 2 was adjusted for age, sex, smoking and systolic blood pressure (SBP); and Model 3 was adjusted for age, sex, smoking, SBP, high-density lipoprotein cholesterol (HDL-C), low-density lipoprotein cholesterol (LDL-C), ACS and revascularization in hospital. The receiver operating characters (ROC) curve analysis was performed to assess the sensitivity and specificity of the hs-CRP for predicting MACCEs (Fig. 2). Additional file 2: Figure S1 shows the comparison of the ROC curves of hs-CRP and lipoprotein(a) for predicting MACCE. The Concordance index, net reclassification improvement (NRI), and integrated discrimination improvement (IDI) were calculated to evaluate the added effect of hs-CRP and the baseline model with established traditional risk factors on the discrimination of clinical outcomes. Established traditional risk factors were age, gender, smoking, hypertension, dyslipidemia and CKD. NRI indicates how many patients improved the accuracy of predicted probability for outcomes. IDI was assessed to determine whether to improve the prediction model. P-values < 0.05 were considered statistically significant. Analyses were carried out using SPSS 24.0, Stata 15 and R 4.1.3.

## Results

### Characteristics of the study patients

A total of 4308 (31.0%) patients were with TVD. After exclusion, 1040 (38%) patients with type 2 DM and 1694 (62%) patients without type 2 DM were eligible for the study. The baseline characteristics of the included patients are detailed in Table 1. 268 (8.7%) patients were treated with medication and 2496 (91.3%) patients underwent revascularization. Of the revascularizations, 2420 (97%) patients underwent PCI and 76 (3%) patients were treated with CABG. Hs-CRP levels were higher in patients with ACS than in those with stable CAD [2.4 (0.9–8.0) vs. 1.7 (0.7–4.9), p < 0.001]. Patients with type 2 DM were predominantly female and were associated with higher rates of comorbidities, including hypertension, dyslipidemia, and CKD, history of MI and cerebrovascular disease, and higher Synergy between Percutaneous Coronary Intervention with Taxus and Cardiac Surgery (SYNTAX) score but lower left ventricular ejection fractions (LVEF). The prevalence of in-hospital death was higher in the DM group than the non-DM group.

**Table 1:** Baseline clinical, angiographic, and procedural characteristics and in-hospital outcome of all patients with and without diabetes before and after stratification according to hs-CRP

Variables	Total population			Patients with diabetes			Patients without diabetes		
	Diabetes	Non-diabetes	P	Hs-CRP < 2 mg/L	Hs-CRP ≥ 2 mg/L	P	Hs-CRP < 2 mg/L	Hs-CRP ≥ 2 mg/L	P
	(n = 1040)	(n = 1694)		(n = 564)	(n = 476)		(n = 812)	(n = 882)	
Age, years	65.3 ± 7.9	64.0 ± 10.4	0.001	65.3 ± 9.4	65.3 ± 10.0	0.769	63.6 ± 10.0	64.4 ± 10.7	0.052
Male	719 (69.1)	1328 (78.4)	< 0.001	401 (71.1)	318 (66.8)	0.135	643 (79.2)	687 (77.7)	0.447
Smoking	381 (36.6)	771 (45.5)	< 0.001	189 (33.5)	192 (40.3)	0.023	336 (41.4)	435 (49.3)	0.001
Hypertension	776 (74.6)	1062 (62.7)	< 0.001	425 (75.4)	351 (73.7)	0.551	478 (58.9)	584 (66.6)	0.002
Dyslipidemia	850 (81.7)	1318 (77.8)	0.014	456 (80.9)	394 (82.8)	0.191	617 (76.0)	701 (79.5)	0.084
CKD	116 (11.2)	144 (8.5)	0.022	42 (7.4)	74 (15.5)	< 0.001	46 (5.7)	98 (11.1)	< 0.001
Prior MI	50 (4.8)	55 (3.2)	0.039	16 (2.8)	34 (7.1)	0.001	28 (3.4)	27 (3.1)	0.653
Prior PAD	40 (3.8)	46 (2.7)	0.100	19 (3.4)	21 (4.4)	0.384	18 (2.2)	28 (3.2)	0.226
Prior cerebrovascular disease	157 (14.1)	199 (11.7)	0.012	66 (11.7)	80 (16.8)	0.018	93 (11.5)	84 (9.5)	0.195
SBP, mm Hg	139 ± 22	137 ± 23	0.006	139 ± 21	139 ± 23	0.943	138 ± 21	137 ± 24	0.369
DBP, mm Hg	80 ± 23	81 ± 13	0.005	79 ± 10	80 ± 32	0.707	81 ± 13	80 ± 14	0.282
Hs-CRP, mg/L	2.1 (0.7–5.9)	1.8 (0.8–5.6)	0.116	0.8 (0.5–1.4)	2.3 (0.8–5.9)	< 0.001	0.8 (0.5–1.2)	1.9 (0.7–5.1)	< 0.001
WBC, 10^9^/L	7.78 ± 2.83	7.71 ± 2.57	0.849	7.41 ± 2.73	8.21 ± 2.90	< 0.001	7.33 ± 2.42	8.06 ± 2.66	< 0.001
FBG, mmol/L	10.3 ± 5.0	6.0 ± 2.0	< 0.001	9.5 ± 4.8	11.3 ± 5.1	< 0.001	5.8 ± 1.7	6.3 ± 2.3	< 0.001
HbA1c, %	8.5 ± 1.7	6.2 ± 1.2	0.007	8.3 ± 1.6	8.6 ± 1.8	0.042	6.2 ± 1.3	6.2 ± 1.2	0.315
TC, mmol/L	4.86 ± 1.18	5.04 ± 1.25	0.031	4.81 ± 1.13	4.94 ± 1.25	0.392	4.84 ± 1.22	5.27 ± 1.25	< 0.001
Triglyceride, mmol/L	2.13 ± 1.51	1.76 ± 1.15	< 0.001	2.05 ± 1.18	2.26 ± 1.92	0.773	1.62 ± 1.04	1.93 ± 1.25	< 0.001
HDL-C, mmol/L	1.23 ± 0.31	1.24 ± 0.31	0.787	1.21 ± 0.30	1.26 ± 0.33	0.132	1.21 ± 0.30	1.27 ± 0.31	0.017
LDL-C, mmol/L	2.85 ± 0.79	3.00 ± 0.89	0.011	2.82 ± 0.74	2.89 ± 0.87	0.716	2.87 ± 0.88	3.15 ± 0.88	< 0.001
Creatinine clearance, mL/min	94 ± 29	89 ± 23	< 0.001	96 ± 27	91 ± 30	0.021	91 ± 21	88 ± 25	0.032
LVEF, %	52.7 ± 9.2	54.2 ± 7.9	< 0.001	53.4 ± 8.8	51.8 ± 9.6	< 0.001	55.0 ± 7.2	53.4 ± 8.5	< 0.001
Acute coronary syndrome	364 (35.0)	604 (35.7)	0.728	182 (32.3)	182 (38.2)	0.044	247 (30.4)	357 (40.5)	< 0.001
Revascularization in hospital	937 (90.1)	1559 (92.0)	0.082	509 (90.2)	428 (89.9)	0.858	755 (93.0)	804 (91.1)	0.166
LMT involvement	159 (15.3)	218 (12.9)	0.075	98 (17.4)	61 (12.8)	0.042	105 (12.9)	113 (12.8)	0.942
SYNTAX score	26.1 ± 9.8	24.7 ± 9.6	0.013	25.9 ± 9.9	26.2 ± 10.1	0.614	24.5 ± 9.8	24.9 ± 9.9	0.257
DES used	821 (78.9)	1423 (84.0)	0.001	450 (79.8)	371 (77.9)	0.467	702 (86.5)	721 (81.7)	0.008
In-hospital death	14 (1.3)	5 (0.3)	0.001	5 (0.9)	9 (1.9)	0.161	2 (0.2)	3 (0.3)	0.722

*CKD* chronic kidney disease, *DBP* diastolic blood pressure, *DES* drug-eluting stent, *FBG* fasting blood glucose, *HbA1c* glycated hemoglobin, *HDL-C* high-density lipoprotein cholesterol, *Hs-CRP* high-sensitivity C-reactive protein, *LDL-C* low-density lipoprotein cholesterol, *LMT* left main trunk, *LVEF* left ventricular ejection fraction, *MI* myocardial infarction, *PAD* peripheral artery disease, *SBP* systolic blood pressure, *SYNTAX* Synergy between Percutaneous Coronary Intervention with Taxus and Cardiac Surgery, *TC* total cholesterol, *WBC* white blood cell

In the diabetic group, patients with high hs-CRP had a significantly higher incidence of smoking, CKD, ACS, prior MI, prior cerebrovascular disease, LMT involvement as well as lower LVEF on admission. No significant differences in the age, hypertension, SBP, LDL-C, in-hospital death rates were found between the high and low hs-CRP groups.

In the non-diabetic group, patients with high hs-CRP were more likely to have smoking, hypertension, ACS, CKD, and higher concentrations of lipid profile. In addition, LVEF was higher in patients in the low hs-CRP group. We did not observe significant differences in dyslipidemia, prior MI, SBP and in-hospital death rates.

### Association between hs-CRP and outcomes

**Fig. 3:**
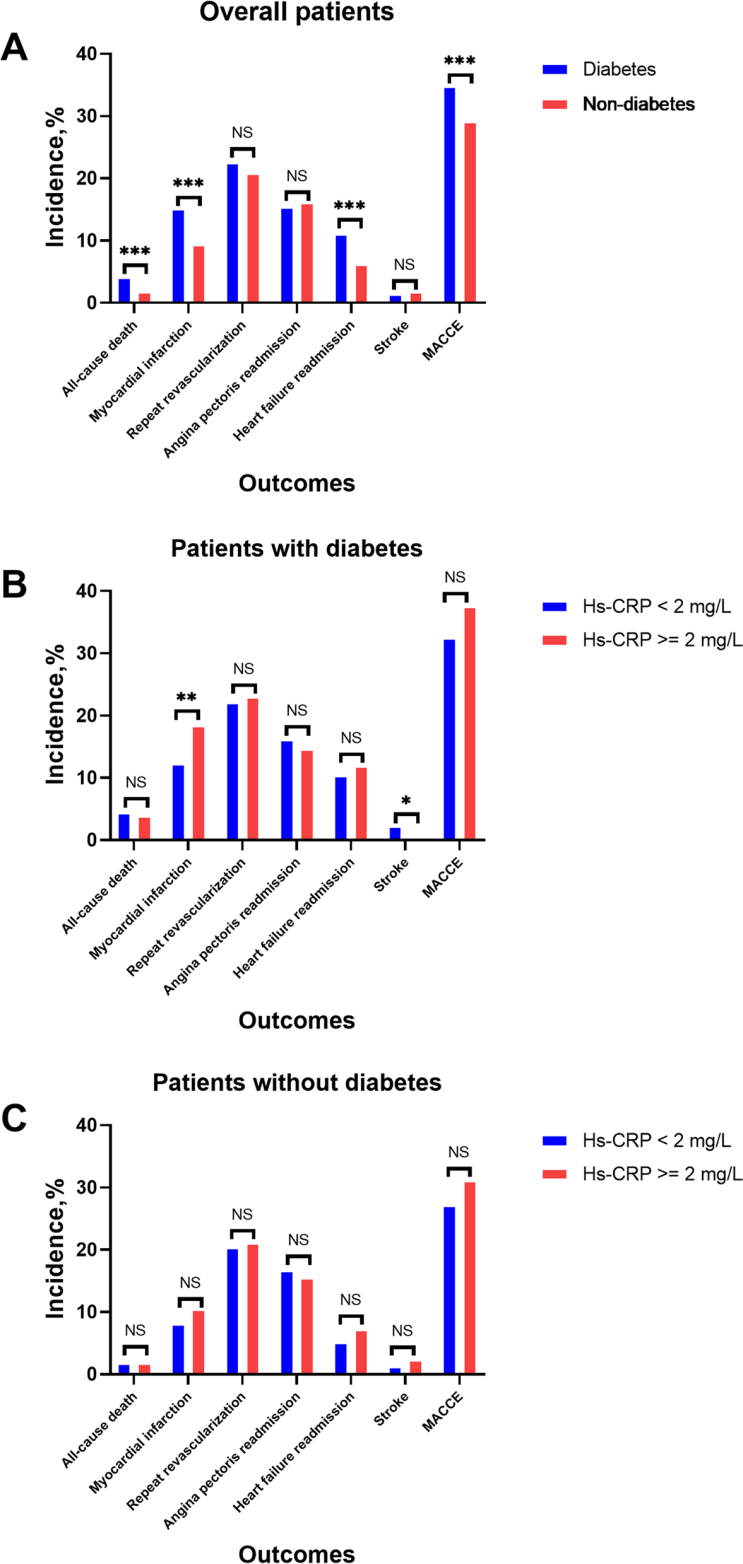
Clinical outcomes of all patients with and without diabetes before (**A**) and after stratification according to hs-CRP levels (**B** and **C**). *P < 0.05. **P < 0.01. ***P < 0.001. *Hs-CRP* high-sensitivity C-reactive protein, *MACCE* major adverse cardiac and cerebrovascular events, *NS* no significance

**Fig. 4:**
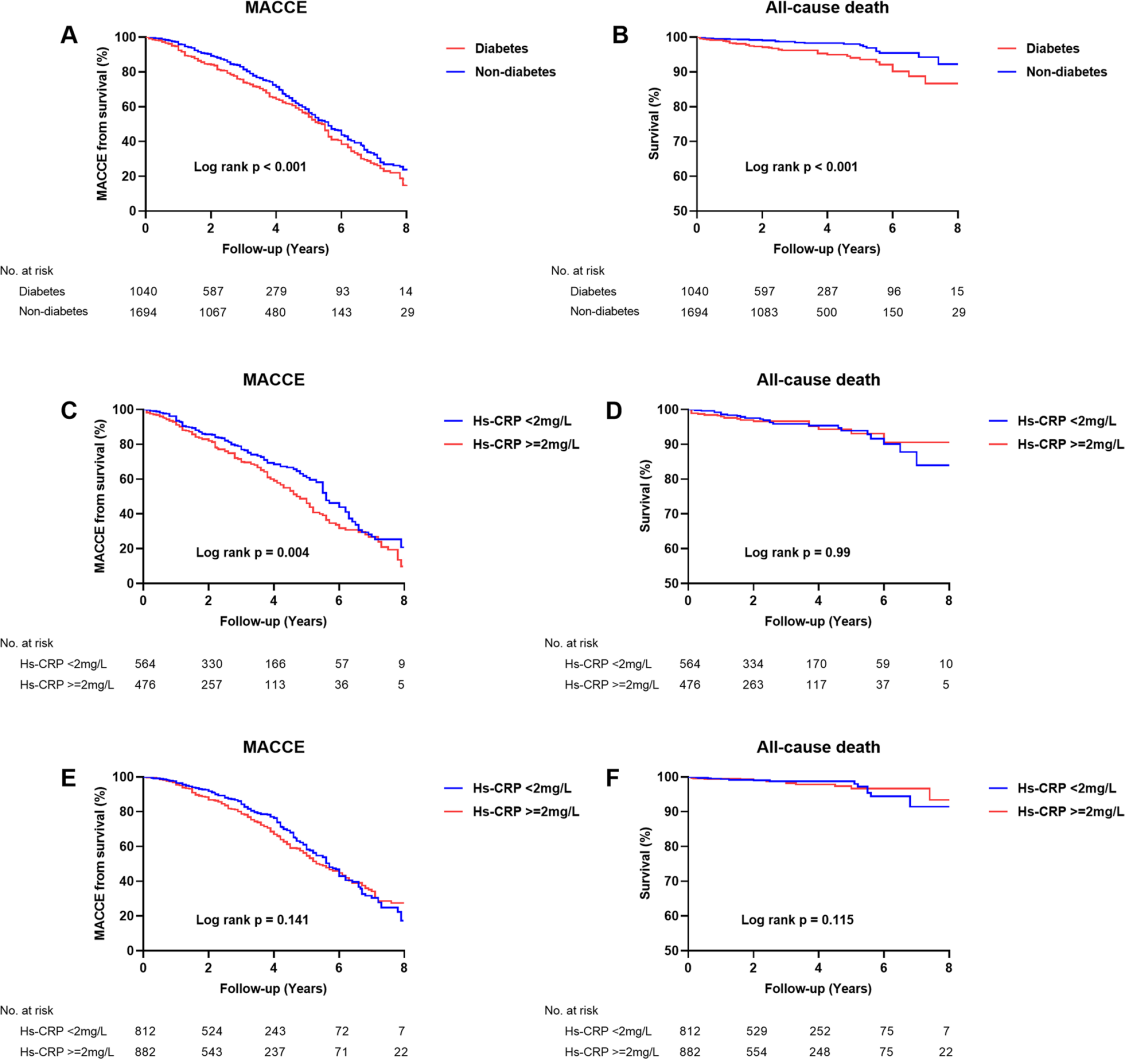
Kaplan-Meier curves for MACCE and all-cause death during follow-up for patients in each subgroup. **A** and **B**: Kaplan-Meier curves for MACCE (**A**) and all-cause death (**B**) during follow-up for all patients according to diabetes; **C** and **D**: Kaplan-Meier curves for MACCE (**C**) and all-cause death (**D**) during follow-up for patients with diabetes according to hs-CRP levels; Figure E and F: Kaplan-Meier curves for MACCE (**E**) and all-cause death (**F**) during follow-up for patients without diabetes according to hs-CRP levels; *Hs-CRP* high-sensitivity C-reactive protein, *MACCE* major adverse cardiac and cerebrovascular events

The median follow-up was 2.4 (1.1–4.1) years. The incidence of clinical outcomes is shown in Fig. 3. Multivariate Cox regression analyses showed that the incidence of MACCE (adjusted hazard ratio [HR] 1.17, 95% confidence interval [CI] 1.01–1.35, p = 0.031), all-cause death (HR 1.82, 95% CI 1.07–3.11, p = 0.026), MI (HR 1.56, 95% CI 1.23–1.98, p < 0.001) and heart failure readmission (HR 1.49, 95% CI 1.11–1.99, p = 0.007) were significantly higher in the diabetic group compared to the non-diabetic group (Table 2) (Fig. 4).

**Table 2:** Clinical outcomes of all patients with TVD stratified according to diabetes mellitus

	Diabetes (n = 1040)	Non-diabetes (n = 1694)	Adjusted HR (95% CI)^*^	P
All-cause death	42 (4.0)	29 (1.7)	1.82 (1.07–3.11)	0.026
Myocardial infarction	156 (15.0)	156 (9.2)	1.56 (1.23–1.98)	< 0.001
Repeat revascularization	233 (22.4)	350 (20.7)	1.10 (0.92–1.31)	0.265
Angina pectoris readmission	159 (15.3)	271 (16.0)	0.99 (0.81–1.23)	0.998
Heart failure readmission	114 (11.0)	104 (6.1)	1.49 (1.11–1.99)	0.007
Stroke	14 (1.3)	28 (1.7)	0.67 (0.33–1.37)	0.279
MACCE	361 (34.7)	492 (29.0)	1.17 (1.01–1.35)	0.031

*Covariates were adjusted for age, sex, smoking, hypertension, dyslipidemia, chronic kidney disease, prior myocardial infarction, left ventricular ejection fraction, ACS and revascularization in hospital

*ACS* acute coronary syndrome, *CI* confidence interval(s), *HR* hazard ratio, *MACCE* major adverse cardiac and cerebrovascular events, *TVD* three-vessel disease

For DM patients, compared with the low hs-CRP group, the high hs-CRP group was strongly related to increases in MACCE (HR: 1.51; 95% CI 1.09–2.10, p = 0.013) after adjusting for age, sex, smoking, SBP, HDL-C, LDL-C, ACS and revascularization in-hospital according to the multivariable Model 3 (Table 3) (Fig. 4). Moreover, the association between MACCE and hs-CRP remained significant in Model 1 and Model 2. However, no significant differences were found for all-cause death (HR 1.63; 95% CI 0.58–4.58, p = 0.349), angina pectoris readmission (HR 1.32; 95% CI 0.81–2.14, p = 0.262), heart failure readmission (HR 1.27; 95% CI 0.69–2.34, p = 0.438) and stroke (HR 0.87; 95% CI 0.08–9.22, p = 0.910) except for MI (HR 2.33; 95% CI 1.34–4.05, p = 0.002) and repeat revascularization (HR 1.71; 95% CI 1.13–2.60, p = 0.011) in Model 3 (Additional file 1: Table S1).

**Table 3:** Association between hs-CRP and endpoints in TVD patients with and without diabetes

Patients with diabetes	Events (n%)	Model 1^a^		Model 2^b^		Model 3^c^	
		HR (95% CI)	P	HR (95% CI)	P	HR (95% CI)	P
All-cause death							
Hs-CRP < 2 mg/L	24 (4.3)	Reference		Reference		Reference	
Hs-CRP ≥ 2 mg/L	18 (3.8)	0.99 (0.54–1.84)	0.997	1.03 (0.55–1.92)	0.924	1.63 (0.58–4.58)	0.349
MACCE							
Hs-CRP < 2 mg/L	183 (32.4)	Reference		Reference		Reference	
Hs-CRP ≥ 2 mg/L	178 (37.4)	1.34 (1.09–1.65)	0.005	1.29 (1.05–1.60)	0.015	1.51 (1.09–2.10)	0.013

Patients without diabetes	Events (n%)	Model 1^a^		Model 2^b^		Model 3^c^	
		HR (95% CI)	P	HR (95% CI)	P	HR (95% CI)	P
All-cause death							
Hs-CRP < 2 mg/L	14 (1.7)	Reference		Reference		Reference	
Hs-CRP ≥ 2 mg/L	15 (1.7)	0.89 (0.42–1.85)	0.757	0.82 (0.38–1.74)	0.606	0.22 (0.05–0.93)	0.039
MACCE							
Hs-CRP < 2 mg/L	219 (27.0)	Reference		Reference		Reference	
Hs-CRP ≥ 2 mg/L	273 (31.0)	1.14 (0.95–1.36)	0.145	1.15 (0.96–1.38)	0.116	0.93 (0.71–1.22)	0.613

^a^Model 1: covariates were adjusted for age and sex

^b^Model 2: covariates were adjusted for age, sex, smoking and SBP

^c^Model 3: covariates were adjusted for age, sex, smoking, SBP, HDL-C, LDL-C, ACS and revascularization in hospital

*ACS* acute coronary syndrome, *CI* confidence interval(s), *HDL-C* high-density lipoprotein cholesterol, *HR* hazard ratio, *Hs-CRP* high-sensitivity C-reactive protein, *LDL-C* low-density lipoprotein cholesterol, *MACCE* major adverse cardiac and cerebrovascular events, *PCI* percutaneous coronary intervention, *SBP* systolic blood pressure, *TVD* three-vessel disease

In non-DM patients, the difference of MACCE (HR 0.93, 95% CI 0.71–1.22, p = 0.613) rate was not observed between the high and low hs-CRP groups in the multivariable Models 1, 2 or 3 (Table 3) (Fig. 4). Notably, the prevalence of other clinical outcomes, including repeat revascularizations, angina pectoris readmissions, heart failure readmissions, and nonfatal strokes, were not significantly different between the two groups in all multivariable Models, except for all-cause death in Model 3 (Additional file1: Table S1).

### Adding hs-CRP to the baseline model for the prediction of outcomes

In the diabetic group, regarding discrimination, MACCE (p = 0.008) reached statistical significance in the C-index when combining hs-CRP with traditional risk factors, while all-cause death (p = 0.510), repeat revascularization (p = 0.102) angina pectoris readmission (p = 0.091), heart failure readmission (p = 0.245) and stroke (p = 0.152) showed a nonsignificant change, except for MI (p = 0.04) (Table 4) (Additional file 1: Table S2). The addition of hs-CRP to the baseline model also significantly increased the NRI (0.2074, p = 0.001) and IDI (0.0086, p = 0.003) of MACCE.

**Table 4:** Evaluation of predictive models for endpoints using the C-index, NRI and IDI

Patients with diabetes	C-index (95% CI)	P	NRI (95% CI)	P	IDI (95% CI)	P
All-cause death						
Traditional risk factors	0.68 (0.60–0.77)	Reference	Reference	–	Reference	–
Traditional risk factors + hs-CRP	0.70 (0.61–0.79)	0.510	0.1314 (−0.1648–0.4276)	0.384	6e-04 (−0.0011–0.0022)	0.504
MACCE						
Traditional risk factors	0.53 (0.50–0.57)	Reference	Reference	–	Reference	–
Traditional risk factors + hs-CRP	0.59 (0.56–0.63)	0.008	0.2074 (0.0804–0.3344)	0.001	0.0086 (0.003–0.0142)	0.003

Patients without diabetes	C-index (95% CI)	P	NRI (95% CI)	P	IDI (95% CI)	P
All-cause death						
Traditional risk factors	0.74 (0.64–0.84)	Reference	Reference	–	Reference	–
Traditional risk factors + hs-CRP	0.76 (0.65–0.86)	0.287	0.0883 (−0.2087–0.3853)	0.560	0.0054 (−0.0026–0.0133)	0.185
MACCE						
Traditional risk factors	0.52 (0.49–0.55)	Reference	Reference	–	Reference	–
Traditional risk factors + hs-CRP	0.56 (0.53–0.59)	0.018	0.0988 (−0.0052–0.2027)	0.062	0.0031 (2e-04–0.006)	0.036

Traditional risk factors included age, gender, smoking, hypertension, hyperlipidemia and chronic kidney disease

*C-index* concordance index, *CI* confidence interval, *HR* hazard ratio, *Hs-CRP* high-sensitivity C-reactive protein, *IDI* integrated discrimination improvement, *NRI* net reclassification improvement, *MACCE* major adverse cardiac and cerebrovascular events

In the non-diabetic group, the C-index for MACCE (p = 0.018) was greater in the baseline model with hs-CRP compared with the traditional risk factors. However, the NRI (0.0988, p = 0.062) for MACCE was not significantly increased after hs-CRP was added to the traditional risk factors (Table 4). The C-index, NRI and IDI for all-cause death, MI, repeat revascularization, angina pectoris readmission, heart failure readmission and stroke are listed in Additional file 1:Table S2.

## Discussion

The present study demonstrated that elevated hs-CRP was associated with MACCE in type 2 diabetic patients but not in non-DM patients with TVD. To the best of our knowledge, this study is the first to specifically investigate the role of hs-CRP in predicting long-term clinical outcomes in a large cohort of TVD patients with and without type 2 diabetes.

Among CAD patients, those with TVD were most susceptible to cardiovascular adverse events. It is widely acknowledged that TVD is characterized by a persistent low-grade inflammatory state [5]. Furthermore, patients with TVD exhibit a more prominent inflammatory state compared to single vessel disease [15]. Current evidence suggests that inflammation plays a key role in all phases of coronary atherothrombosis, including plaque progression, rupture, and thrombosis causing acute MI. Possible mechanisms of low-grade inflammation-induced atherosclerosis include accelerated foam cell formation and local LDL-C uptake in the arterial wall [16].

CRP is synthesized and secreted in the liver 6 h after an acute inflammatory stimulus [17]. CRP is positively associated with the metabolic syndrome and may be an independent risk factor in patients with CAD. It is thought that CRP inhibits endothelial nitric oxide production and promotes the recruitment of monocytes into atheromatous plaques by increasing the expression of endothelial cell adhesion molecules, and leading to plaque instability [18, 19].

An increasing body of evidence suggests that hs-CRP levels above 2 mg/L indicate residual inflammatory risk [11, 20, 21]. Interestingly, an association between higher plasma levels of hs-CRP and cardiovascular events has been documented in patients with type 2 diabetes [22–24]. Indeed, among patients with non-diabetes, high CRP is associated with the development of type 2 diabetes [25]. A prior study demonstrated that CRP was related to mortality in DM and non-DM patients with acute MI [26]. However, previous studies on the association between hs-CRP and long-term outcomes in TVD patients with type 2 diabetes are scanty, and the prognostic value of hs-CRP in this patient population remains unknown.

In this study, we substantiated that elevated hs-CRP had a prognostic value for MACCE in DM patients but not in non-DM patients with TVD. Three multivariable analysis models were performed to elevate the independent effects of the hs-CRP and clinical outcomes of TVD patients with or without type 2 diabetes after adjusting for other potential confounders. The association remained significant in TVD patients with type 2 diabetes even after adjustment for other independent variables. The addition of hs-CRP to the established risk model improved the predictive power (C-index) for adverse cardiovascular events in diabetic patients. Additionally, it enabled stratification into different risk categories based on the NRI and IDI. Taken together, our findings suggest the inflammatory status revealed by hs-CRP is a valid parameter for predicting MACCE in patients with TVD with diabetes. To the best of our knowledge, this is the first study to reveal the good predictive value of hs-CRP for MACCEs in TVD patients with type 2 diabetes.

Regarding the relationship between inflammation and clinical outcomes, hs-CRP was associated with different outcomes in DM and non-DM patients in our study, which may be attributed to DM being a multifactorial metabolic disease characterized by a state of sub-clinical inflammation [6, 27]. Moreover, DM is more frequently associated with some degree of chronic inflammation, which is reflected by chronically high levels of hs-CRP. Overall, both type 2 diabetes and atherosclerosis are multifactorial diseases and may have a common inflammatory basis [28]. Elevated hs-CRP may promote insulin resistance through the production of proinflammatory cytokines such as interleukin-1β, interacting with adipose tissue-specific macrophages and activation of innate immune system [25]. Notably, older patients, hypertension, dyslipidemia, CKD, lower LVEF and higher SYNTAX score were less common in the non-diabetic group. That means patients with non-diabetes exhibited fewer risk factors and chronic inflammation compared to those with diabetes, and together, therefore, these factors may reduce the impact of hs-CRP on clinical outcomes.

Aspirin and statins have been documented to indirectly decrease thrombosis, hs-CRP and inflammation, accounting for their use clinically to reduce cardiovascular events [29, 30]. In the recent CANTOS study, anti-inflammatory therapy with canakinumab targeting the interleukin-1β innate immunity pathway could significantly reduce plasma levels of hs-CRP and the risk of recurrent cardiovascular events in patients with previous MI and hs-CRP level > 2 mg/L, without interfering with lipid levels [31]. Interestingly, a post hoc analysis showed that among approximately 4,000 patients with normalized hs-CRP levels after the first dose, canakinumab resulted in a much larger reduction of events, including a 30% reduction in overall mortality [32]. The above findings suggest that hs-CRP-lowering treatment has huge prospects as an innovative approach to treating CAD patients.

The effectiveness of anti-inflammatory drugs given prior to PCI remains controversial. Our study has demonstrated that inflammatory states predispose to poor outcomes in type 2 diabetic patients with TVD, suggesting that targeting hs-CRP may also have a role in secondary prevention in those high-risk patients. Nevertheless, the question of whether hs-CRP can be used as a target marker for statin therapy remains controversial. Further basic experiments and clinical trials are necessary to clarify the potential of hs-CRP.

### Study limitations

This study has several limitations. First, this study is limited by its observational nature. There is heterogeneity in the study population and it may affect clinical outcomes. Second, although hs-CRP is a nonspecific biomarker linked with multiple clinical conditions, multivariable Cox regression analysis models performed in the present study confirmed the constant association between hs-CRP and MACCE. Third, we recorded hs-CRP only once on admission and did not record changes in hs-CRP during follow-up. Fourth, the predictive value of hs-CRP for patients with single vessel disease or two vessel disease was not evaluated. Nevertheless, the results of our large cohort study indicate that hs-CRP could be a useful biomarker for predicting the risk of adverse cardiovascular events in TVD patients with type 2 diabetes.

## Conclusions

Elevated hs-CRP was associated with an increased risk of MACCE in type 2 diabetic patients with TVD but not in non-type 2 DM patients with TVD. Compared to traditional risk factors, hs-CRP improved the risk prediction of adverse cardiovascular events in TVD patients with type 2 diabetes. Nonetheless, well-designed and randomized controlled trials are warranted to verify the relationship between hs-CRP and cardiovascular outcomes in this patient population.

## Supplementary Information

**Additional** **file 1: Table S1.** Association between hs-CRP and adverse events in TVD patients with and without diabetes. **Table S2.** Evaluation of predictive models for adverse events using the C-index, NRI and IDI.

**Additional file** **2:  Figure S1.** Comparison of the ROC curves of hs-CRP and lipoprotein(a) for predicting MACCE. *AUC* area under the curve, *Hs-CRP* high-sensitivity C-reactive protein, *Lp(a)* lipoprotein(a), *MACCE* major adverse cardiac and cerebrovascular events, *ROC* receiver operating curve.

## Data Availability

The datasets generated and analyzed for this current study are available from the corresponding author upon reasonable request.

## Abbreviations

CAD: Coronary artery disease
DM: Diabetes mellitus
Hs-CRP: High-sensitivity C-reactive protein
IDI: Integrated discrimination improvement
MACCE: Major adverse cardiac and cerebrovascular events
MI: Myocardial infarction
NRI: Net reclassification improvement
PCI: Percutaneous coronary intervention
TVD: three-vessel disease
