# Supplementary material for: Predictive value of high sensitivity C-reactive protein in three-vessel disease patients with and without type 2 diabetes
Source: Cardiovasc Diabetol. 2023 Apr 20;22:91. doi: 10.1186/s12933-023-01830-7 (PMC10120230; doi:10.1186/s12933-023-01830-7)
Supplement: Supplementary file 1 — Additional file 1: Table S1. Association between hs-CRP and adverse events in TVD patients with and without diabetes. Table S2. Evaluation of predictive models for adverse events using the C-index, NRI and IDI. [file 12933_2023_1830_MOESM1_ESM.docx]

**Table S1 Association between hs-CRP and adverse events in TVD patients with and without diabetes**

| **Patients with diabetes** |  | **Model 1^a^** |  | **Model 2^b^** |  | **Model 3^c^** |  |
| --- | --- | --- | --- | --- | --- | --- | --- |
|  | **Events (n%)** | **HR (95% CI)** | **P** | **HR (95% CI)** | **P** | **HR (95% CI)** | **P** |
| Myocardial infarction |  |  |  |  |  |  |  |
| Hs-CRP <2mg/L | 69 (12.2) | Reference |  | Reference |  | Reference |  |
| Hs-CRP ≥2mg/L | 87 (18.3) | 1.74 (1.27-2.39) | 0.001 | 1.73 (1.25-2.39) | 0.001 | 2.33 (1.34-4.05) | 0.002 |
| Repeat revascularization |  |  |  |  |  |  |  |
| Hs-CRP <2mg/L | 124 (22.0) | Reference |  | Reference |  | Reference |  |
| Hs-CRP ≥2mg/L | 109 (22.9) | 1.25 (0.96-1.62) | 0.087 | 1.21 (0.93-1.57) | 0.149 | 1.71 (1.13-2.60) | 0.011 |
| Angina pectoris readmission |  |  |  |  |  |  |  |
| Hs-CRP <2mg/L | 90 (16.0) | Reference |  | Reference |  | Reference |  |
| Hs-CRP ≥2mg/L | 69 (14.5) | 1.11 (0.81-1.52) | 0.510 | 1.04 (0.76-1.44) | 0.771 | 1.32 (0.81-2.14) | 0.262 |
| Heart failure readmission |  |  |  |  |  |  |  |
| Hs-CRP <2mg/L | 58 (10.3) | Reference |  | Reference |  | Reference |  |
| Hs-CRP ≥2mg/L | 56 (11.8) | 1.30 (0.90-1.88) | 0.157 | 1.25 (0.85-1.81) | 0.245 | 1.27 (0.69-2.34) | 0.438 |
| Stroke |  |  |  |  |  |  |  |
| Hs-CRP <2mg/L | 12 (2.1) | Reference |  | Reference |  | Reference |  |
| Hs-CRP ≥2mg/L | 2 (0.4) | 0.21 (0.05-0.97) | 0.046 | 0.22 (0.05-1.02) | 0.054 | 0.87 (0.08-9.22) | 0.910 |

| **Patients without diabetes** |  | **Model 1^a^** |  | **Model 2^b^** |  | **Model 3^c^** |  |
| --- | --- | --- | --- | --- | --- | --- | --- |
|  | **Events (n%)** | **HR (95% CI)** | **P** | **HR (95% CI)** | **P** | **HR (95% CI)** | **P** |
| Myocardial infarction |  |  |  |  |  |  |  |
| Hs-CRP <2mg/L | 65 (8.0) | Reference |  | Reference |  | Reference |  |
| Hs-CRP ≥2mg/L | 91 (10.3) | 1.20 (0.87-1.66) | 0.253 | 1.22 (0.88-1.69) | 0.227 | 0.88 (0.53-1.44) | 0.616 |
| Repeat revascularization |  |  |  |  |  |  |  |
| Hs-CRP <2mg/L | 165 (20.3) | Reference |  | Reference |  | Reference |  |
| Hs-CRP ≥2mg/L | 185 (21.0) | 1.03 (0.83-1.27) | 0.756 | 1.05 (0.85-1.31) | 0.602 | 0.79 (0.58-1.09) | 0.160 |
| Angina pectoris readmission |  |  |  |  |  |  |  |
| Hs-CRP <2mg/L | 135 (16.6) | Reference |  | Reference |  | Reference |  |
| Hs-CRP ≥2mg/L | 136 (15.4) | 0.93 (0.73-1.18) | 0.555 | 0.93 (0.73-1.19) | 0.588 | 0.76 (0.53-1.08) | 0.129 |
| Heart failure readmission |  |  |  |  |  |  |  |
| Hs-CRP <2mg/L | 41 (5.0) | Reference |  | Reference |  | Reference |  |
| Hs-CRP ≥2mg/L | 63 (7.1) | 1.35 (0.90-2.01) | 0.137 | 1.29 (0.86-1.93) | 0.212 | 1.00 (0.53-1.86) | 0.998 |
| Stroke |  |  |  |  |  |  |  |
| Hs-CRP <2mg/L | 9 (1.1) | Reference |  | Reference |  | Reference |  |
| Hs-CRP ≥2mg/L | 19 (2.2) | 1.91 (0.86-4.25) | 0.112 | 1.98 (0.89-4.43) | 0.093 | 1.24 (0.24-6.23) | 0.787 |

^a^Model 1: covariates were adjusted for age and sex

^b^Model 2: covariates were adjusted for age, sex, smoking and SBP

^c^Model 3: covariates were adjusted for age, sex, smoking, SBP, HDL-C, LDL-C, ACS and revascularization in hospital

*ACS* acute coronary syndrome, *CI* confidence interval(s), *HDL-C* high-density lipoprotein cholesterol, *HR* hazard ratio, *Hs-CRP* high-sensitivity C-reactive protein, *LDL-C* low-density lipoprotein cholesterol, *PCI* percutaneous coronary intervention, *SBP* systolic blood pressure, *TVD* three-vessel disease

**Table S2 Evaluation of predictive models for adverse events using the C-index, NRI and IDI**

| **Patients with diabetes** | **C-index (95% CI)** | **P** | **NRI (95% CI)** | **P** | **IDI (95% CI)** | **P** |
| --- | --- | --- | --- | --- | --- | --- |
| Myocardial infarction |  |  |  |  |  |  |
| Traditional risk factors | 0.57 (0.53-0.62) | Reference | Reference | - | Reference | - |
| Traditional risk factors + hs-CRP | 0.62 (0.57-0.66) | 0.040 | 0.1388 (-0.0089 - 0.2864) | 0.065 | 0 (-1e-04 - 1e-04) | 0.617 |
| Repeat revascularization |  |  |  |  |  |  |
| Traditional risk factors | 0.54 (0.50-0.58) | Reference | Reference | - | Reference | - |
| Traditional risk factors + hs-CRP | 0.57 (0.54-0.62) | 0.102 | 0.0364 (-0.0805 - 0.1533) | 0.541 | 0.0012 (-7e-04 - 0.0032) | 0.203 |
| Angina pectoris readmission |  |  |  |  |  |  |
| Traditional risk factors | 0.58 (0.54-0.63) | Reference | Reference | - | Reference | - |
| Traditional risk factors + hs-CRP | 0.62 (0.57-0.67) | 0.091 | 0.128 (-0.0014 - 0.2574) | 0.052 | 0.0038 (0.0011 - 0.0065) | 0.006 |
| Heart failure readmission |  |  |  |  |  |  |
| Traditional risk factors | 0.59 (0.53-0.64) | Reference | Reference | - | Reference | - |
| Traditional risk factors + hs-CRP | 0.61 (0.56-0.67) | 0.245 | 0.1813 (0.0087 - 0.3538) | 0.039 | 6e-04 (-0.0012 - 0.0024) | 0.492 |
| Stroke |  |  |  |  |  |  |
| Traditional risk factors | 0.72 (0.63-0.81) | Reference | Reference | - | Reference | - |
| Traditional risk factors + hs-CRP | 0.79 (0.69-0.90) | 0.152 | 0.4185 (0.1432 - 0.6939) | 0.003 | 0.001 (-0.0036 - 0.0055) | 0.677 |
|  |  |  |  |  |  |  |
| **Patients without diabetes** | **C-index (95% CI)** | **P** | **NRI (95% CI)** | **P** | **IDI (95% CI)** | **P** |
| Myocardial infarction |  |  |  |  |  |  |
| Traditional risk factors | 0.62 (0.57-0.67) | Reference | Reference | - | Reference | - |
| Traditional risk factors + hs-CRP | 0.62 (0.57-0.67) | 0.334 | 0.0595 (-0.08 - 0.199) | 0.402 | 1e-04 (-5e-04 - 6e-04) | 0.753 |
| Repeat revascularization |  |  |  |  |  |  |
| Traditional risk factors | 0.55 (0.52-0.58) | Reference | Reference | - | Reference | - |
| Traditional risk factors + hs-CRP | 0.57 (0.54-0.61) | 0.124 | -0.036 (-0.1342 - 0.0622) | 0.472 | 0 (2e-04 - 2e-04) | 0.868 |
| Angina pectoris readmission |  |  |  |  |  |  |
| Traditional risk factors | 0.60 (0.57-0.64) | Reference | Reference | - | Reference | - |
| Traditional risk factors + hs-CRP | 0.62 (0.58-0.65) | 0.174 | -0.0029 (-0.1107 - 0.1049) | 0.958 | 1e-04 (-6e-04 - 8e-04) | 0.733 |
| Heart failure readmission |  |  |  |  |  |  |
| Traditional risk factors | 0.65 (0.60-0.71) | Reference | Reference | - | Reference | - |
| Traditional risk factors + hs-CRP | 0.68 (0.63-0.73) | 0.115 | 0.1813 (0.0087 - 0.3538) | 0.039 | 6e-04 (-0.0012 - 0.0024) | 0.492 |
| Stroke |  |  |  |  |  |  |
| Traditional risk factors | 0.71 (0.63-0.78) | Reference | Reference | - | Reference | - |
| Traditional risk factors + hs-CRP | 0.75 (0.67-0.82) | 0.191 | -0.0013 (-0.0037 - 0.0012) | 0.317 | -0.0018 (-0.0032 - -4e-04) | 0.013 |

*C-index* concordance index, *CI* confidence interval, *HR* hazard ratio, *Hs-CRP* high-sensitivity C-reactive protein, *IDI* integrated discrimination improvement, *NRI* net reclassification improvement

Traditional risk factors included age, gender, smoking, hypertension, hyperlipidemia and chronic kidney disease
