# Supplementary figures and images for: Predictive value of high sensitivity C-reactive protein in three-vessel disease patients with and without type 2 diabetes
Source: Cardiovasc Diabetol. 2023 Apr 20;22:91. doi: 10.1186/s12933-023-01830-7 (PMC10120230; doi:10.1186/s12933-023-01830-7)

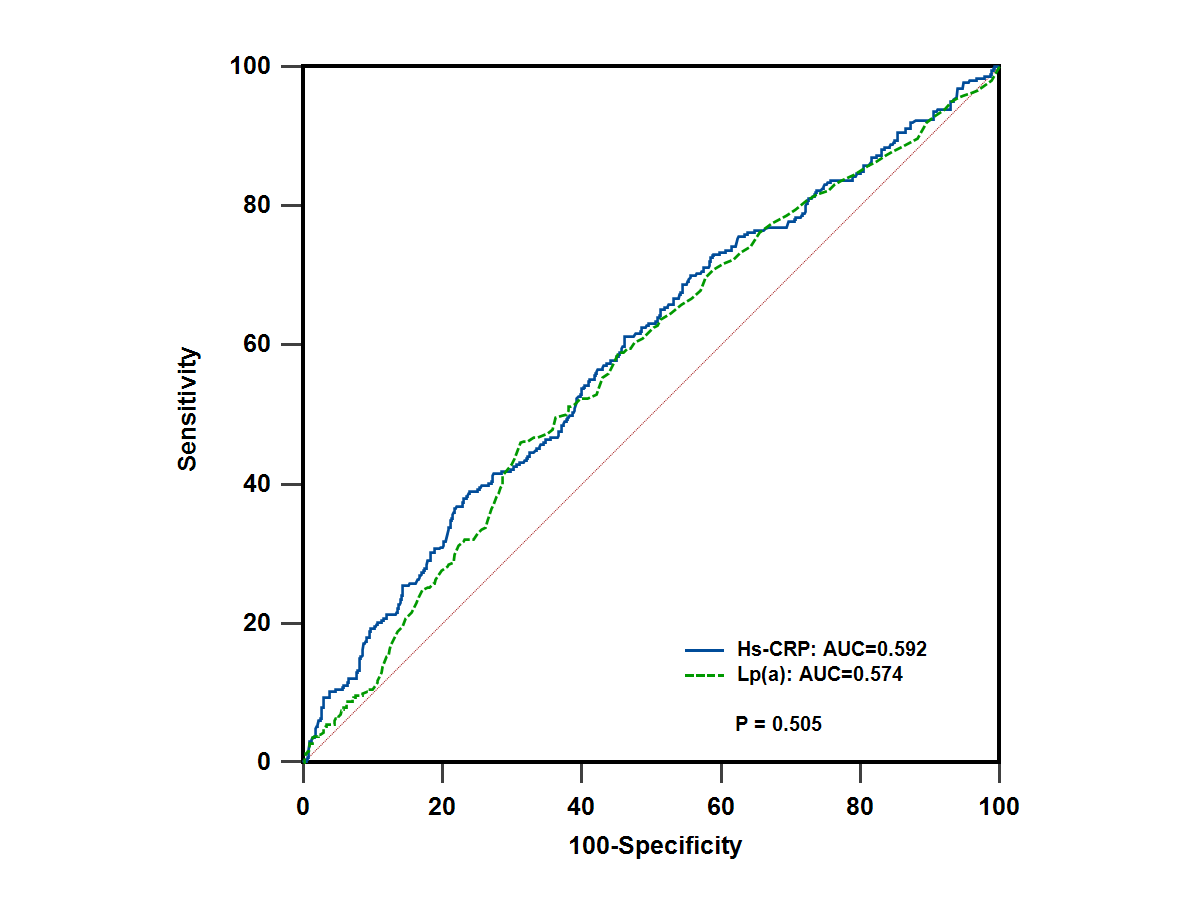

Supplement: Supplementary file 2 — Additional file 2: Figure S1. Comparison of the ROC curves of hs-CRP and lipoprotein(a) for predicting MACCE. AUC area under the curve, Hs-CRP high-sensitivity C-reactive protein, Lp(a) lipoprotein(a), MACCE major adverse cardiac and cerebrovascular events, ROC receiver operating curve. [file 12933_2023_1830_MOESM2_ESM.tif]
